# Dietary Supplementation With Branched Chain Amino Acids to Improve Sleep in Veterans With Traumatic Brain Injury: A Randomized Double-Blind Placebo-Controlled Pilot and Feasibility Trial

**DOI:** 10.3389/fnsys.2022.854874

**Published:** 2022-05-04

**Authors:** Jonathan E. Elliott, Allison T. Keil, Sara Mithani, Jessica M. Gill, Maya E. O’Neil, Akiva S. Cohen, Miranda M. Lim

**Affiliations:** ^1^VA Portland Health Care System, Portland, OR, United States; ^2^Department of Neurology, Oregon Health & Science University, Portland, OR, United States; ^3^National Institutes of Health, National Institute of Nursing Research, Bethesda, MD, United States; ^4^Department of Psychiatry, Oregon Health & Science University, Portland, OR, United States; ^5^Medical Informatics and Clinical Epidemiology, Oregon Health & Science University, Portland, OR, United States; ^6^Perelman School of Medicine, Anesthesiology and Critical Care Medicine, University of Pennsylvania, Philadelphia, PA, United States; ^7^Anesthesiology, Children’s Hospital of Philadelphia, Joseph Stokes Research Institute, Philadelphia, PA, United States; ^8^Department of Behavioral Neuroscience, Oregon Health & Science University, Portland, OR, United States; ^9^Department of Medicine, Division of Pulmonary and Critical Care Medicine, Oregon Health & Science University, Portland, OR, United States; ^10^Oregon Institute of Occupational Health Sciences, Oregon Health & Science University, Portland, OR, United States; ^11^VA Portland Health Care System, National Center for Rehabilitation and Auditory Research, Portland, OR, United States

**Keywords:** traumatic brain injury (TBI), BCAA, sleep, cognition, blood biomarker, dietary supplementation

## Abstract

**Study Objectives:**

Traumatic brain injury (TBI) is associated with chronic sleep disturbances and cognitive impairment. Our prior preclinical work demonstrated dietary supplementation with branched chain amino acids (BCAA: leucine, isoleucine, and valine), precursors to *de novo* glutamate production, restored impairments in glutamate, orexin/hypocretin neurons, sleep, and memory in rodent models of TBI. This pilot study assessed the feasibility and preliminary efficacy of dietary supplementation with BCAA on sleep and cognition in Veterans with TBI.

**Methods:**

Thirty-two Veterans with TBI were prospectively enrolled in a randomized, double-blinded, placebo-controlled trial comparing BCAA (30 g, b.i.d. for 21-days) with one of two placebo arms (microcrystalline cellulose or rice protein, both 30 g, b.i.d. for 21-days). Pre- and post-intervention outcomes included sleep measures (questionnaires, daily sleep/study diaries, and wrist actigraphy), neuropsychological testing, and blood-based biomarkers related to BCAA consumption.

**Results:**

Six subjects withdrew from the study (2/group), leaving 26 remaining subjects who were highly adherent to the protocol (BCAA, 93%; rice protein, 96%; microcrystalline, 95%; actigraphy 87%). BCAA were well-tolerated with few side effects and no adverse events. BCAA significantly improved subjective insomnia symptoms and objective sleep latency and wake after sleep onset on actigraphy.

**Conclusion:**

Dietary supplementation with BCAA is a mechanism-based, promising intervention that shows feasibility, acceptability, and preliminary efficacy to treat insomnia and objective sleep disruption in Veterans with TBI. A larger scale randomized clinical trial is warranted to further evaluate the efficacy, dosing, and duration of BCAA effects on sleep and other related outcome measures in individuals with TBI.

**Clinical Trial Registration:**

[http://clinicaltrials.gov/], identifier [NCT03990909].

## Introduction

Traumatic brain injury (TBI) is common among US Veterans and military service members (Centers for Disease and Control, 2016; Dewan et al., 2019) and often complicated by post-concussive sequelae (Okie, 2005; Hoge et al., 2008) that does not resolve in the weeks or months following injury (Rutherford et al., 1977). Among these post-concussive symptoms, sleep-wake disturbances (Neylan et al., 1998; Ohayon and Shapiro, 2000; Ouellet et al., 2004; Castriotta et al., 2007; Duclos et al., 2014; Sandsmark et al., 2017) are arguably some of the most pervasive and debilitating. The prevalence of sleep disturbances following TBI is 50–70% (Mathias and Alvaro, 2012), which can persist for >20–25 years post-injury (Kempf et al., 2010; Balba et al., 2018; Elliott et al., 2018b,^2020^), and is associated with staggering functional and economic impacts (Colten and Altevogt(eds), 2006). Evidence has shown that sleep disturbances intensify post-concussive symptoms (Nguyen et al., 2017), impact an individual’s ability to cope with these symptoms (Lew et al., 2009), worsen neuropsychiatric symptoms post-TBI (Rao et al., 2014), inhibit full participation in rehabilitation treatment programs (Worthington and Melia, 2006), and delay or completely prevent return to the workforce, community, and family life (Corrigan et al., 2014). Thus, TBI-related sleep disturbances can also compound the issue by potentially lessening the efficacy of rehabilitative therapies targeted at reducing symptoms that impair quality of life (Worthington and Melia, 2006).

Cognitive disturbances, either independent of or secondary to sleep disturbances, are also highly prevalent (60–80%) in the sub-acute/chronic phase of recovery from TBI (Vasterling et al., 2006; Scholten et al., 2012). Impaired cognitive function in TBI is associated with reduced quality of life (Dijkers, 2004; Mahmood et al., 2004; Moldover et al., 2004; Vasterling et al., 2006; Hoge et al., 2008; Bombardier et al., 2010; Boakye et al., 2016), which are both exacerbated by TBI-related sleep disturbances (Boakye et al., 2016). It logically follows that improving sleep and cognitive function in TBI would improve both functional outcomes and quality of life (Wiseman-Hakes et al., 2013).

Standard clinical interventions for sleep for patients with TBI are not without disadvantages. Therapies to treat sleep-wake disturbances in those with TBI have largely been limited by the lack of mechanistic-based, targeted approaches. In response to this gap, we have built a large body of preclinical work over the past 12 years in order to understand the neural mechanisms by which persistent sleep disruption occurs following TBI. Our prior preclinical work has identified that dietary supplementation with branched chain amino acids (BCAA: isoleucine, leucine, and valine) ameliorates both sleep and cognitive impairments in a rodent model of TBI.

Branched chain amino acid are essential amino acids (cannot be synthesized endogenously) that have widespread effects throughout the body. Previous studies have examined the effects of BCAA supplementation in exercise physiology, hepatic pathology, and various neurological and psychiatric disorders (Cerra et al., 1983; Horst et al., 1984; Skeie et al., 1990; Blomstrand et al., 1991; Plauth et al., 1993; Hasseman et al., 1994; Cangiano et al., 1996; Tandan et al., 1996; Richardson et al., 1999; Blomstrand, 2001; James, 2002; Mori et al., 2002; Marchesini et al., 2003; Scarna et al., 2003; Aquilani et al., 2005; Fernstrom, 2005; Ozgultekin et al., 2008). BCAA supplementation can be safely used for an extended duration of time as some studies have evaluated BCAA use for over 2 years (Fernstrom, 2005), and many athletes consume BCAAs as nutritional supplements for years without adverse effects (Knapik et al., 2016). BCAA are believed to have both anti-catabolic as well as anti-nociceptive effects (Wagenmakers, 1992; Manner et al., 1996).

The main role of BCAAs in the central nervous system (CNS) relates to their function as precursors to *de novo* glutamate and thereby GABA (i.e., the main excitatory and inhibitory neurotransmitters, respectively) synthesis (Yudkoff, 1997; Fernstrom, 2005). The majority of dietary BCAAs are not metabolized by the liver and instead freely pass into the systemic circulation, causing plasma concentrations to rise rapidly after ingestion and in proportion to their dose (or content *via* solid food) (Matthews, 2005). BCAAs in the plasma are able to pass through the blood brain barrier by a CNS endothelial cell transporter (Smith et al., 1987). This transporter also moves other amino acids across the blood brain barrier relative to their concentration gradients. Therefore, in the face of increased plasma BCAA concentration, CNS BCAA concentration rises (Hawkins et al., 2006) while the concentration of competing amino acids (e.g., tryptophan, tyrosine, and phenylalanine) decreases (Robertson et al., 1988). These competing amino acids are precursors to other neurotransmitters (i.e., monoamines and catecholamines). Thus, BCAA consumption in isolation rather than in the presence of other amino acids (e.g., in a liquid protein shake or solid food meal) produces changes in overall CNS biochemical composition.

Our preclinical studies using a mouse model of TBI have shown that BCAA supplementation improves sleep-wake disturbances *via* restoration of glutamate within presynaptic nerve terminals contacting orexin neurons in the hypothalamus (Lim et al., 2013; Elliott et al., 2018a). We have also shown that BCAA supplementation restores the balance of excitation to inhibition within the hippocampus and improves hippocampal-dependent memory tasks (Cole et al., 2010; Elkind et al., 2015). Finally, our preclinical work has tested the optimal dosing, duration, and route of administration of BCAA effects in mice (Elkind et al., 2015). Taken together, there is compelling scientific precedent, mechanistic rationale, and human safety data to support the testing of BCAA supplementation for Veterans with TBI. Thus, we performed a small pilot randomized, double-blinded, placebo-controlled study examining the feasibility, acceptability, and preliminary efficacy of BCAA supplementation to improve sleep in Veterans with TBI. Given our preclinical findings showing improved memory after BCAA supplementation, BCAA effects on both subjective and objective cognitive outcome variables were explored. Finally, that BCAA are anti-nociceptive (Manner et al., 1996), BCAA effects on blood-based biomarkers related to inflammation and subjective and objective measures of chronic pain (both elevated in humans with TBI (Balba et al., 2018; Elliott et al., 2018b; Edwards et al., 2020; Pattinson et al., 2020; Peltz et al., 2020), were also explored. We hypothesized that dietary BCAA supplementation would be feasible, acceptable, and demonstrate preliminary efficacy to improve sleep in Veterans with TBI.

## Materials and Methods

**FIGURE 1:**
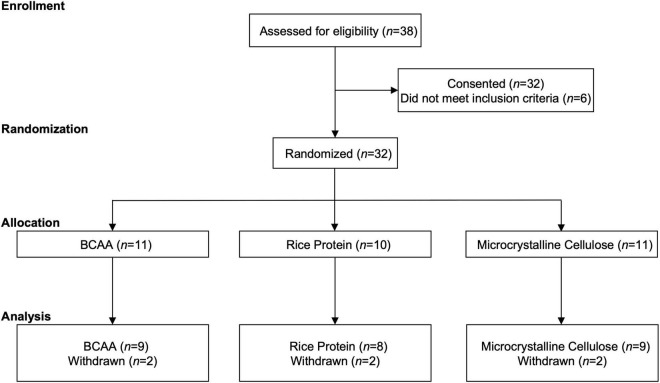
CONSORT. Participant enrollment, randomization, allocation, and analysis following the CONSORT extension for randomized pilot and feasibility guidelines.

The VA Portland Health Care System institutional review board approved this study (IRB #4312), and each participant gave written and verbal informed consent prior to participation in this clinical trial (NCT03990909). Exclusion criteria were, (1) lack of Veteran status, (2) presence of dementia, (3) history of maple syrup urine disease, (4) current supplementation of their diet with BCAAs, and/or (5) not having a prior history of TBI. Participants were screened (*n* = 38) and consented/enrolled (*n* = 32) between 03/2019 and 03/2020. Six participants were excluded due to history of TBI not being confirmed upon further interview. TBI status was determined in-person by a structured clinical interview using the Head Trauma Events Characteristics (HTEC) framework (Cifu et al., 2009), as before (Elliott et al., 2021). The HTEC follows a similar format to other structured clinical interviews [e.g., Ohio State University—OSU TBI ID (Corrigan and Bogner, 2007)], which begins with a standard screening question followed by branching logic questions addressing injury type, location, intracranial injury/skull fracture, duration of loss of consciousness (LOC), and post-traumatic amnesia (PTA; anterograde or retrograde). A diagnosis of no, mild, moderate, or severe TBI, was made and verified by a board-certified neurologist. TBI characteristics in the present study (e.g., age of injury, years since injury) reflect participants self-reported most severe injury, regardless of recency in the event > 1 injury was reported. The remaining *n* = 32 participants were randomized to one of three groups; (1) BCAA (*n* = 11), (2) Rice protein (*n* = 10); or (3) microcrystalline cellulose (*n* = 11). Two of the 3 groups were placebo controls: Rice protein, which controls for total protein content but is low in BCAA content, and microcrystalline cellulose, which is derived from high grade, purified wood pulp and has zero protein content. Two participants from each of the three groups withdrew after enrollment (see Section “Results”). The remaining sample of *n* = 26 participants (BCAA, *n* = 9; Rice, *n* = 8; microcrystalline cellulose, *n* = 9) were included in the present analyses (Figure 1).

Participants began the study adhering to a 2-week baseline period in which they were instructed to consume 20 oz of water directly after waking up, and again 6 h later. At the end of the baseline period, participants were randomized to either BCAA or one of the two placebo groups in a double-blinded manner. Randomization was achieved using a block design stratifying for age (2 blocks: 18–40 years old and 41–65 years old) and sex, performed by the Portland VA Research Pharmacy. Participants then were mailed a 21-day supply consisting of 42 vials that contained 30 g of either BCAA, rice protein, or microcrystalline cellulose to consume b.i.d. (i.e., twice daily) in provided drink shaker bottles (BlenderBottle, Trove Brands, LLC, Lehi, UT, United States) mixed with 20 oz of water. Participants were also provided flavor packets of 5–7 g of Crystal Light Raspberry Lemonade to improve palatability. Participants were instructed on how to prepare and consume their drinks, and to record the date and time they consumed the supplement. Outcomes were assessed at baseline and post-intervention.

### Primary Outcomes

#### Acceptability, Feasibility, and Adherence

Standard measures related to study acceptability, feasibility, and adherence were employed, following the CONSORT extension for randomized pilot and feasibility guidelines (Eldridge et al., 2016a,b; Elkind et al., 2015; Thabane et al., 2016). We specifically focused on several domains: (1) overall study completion rate, (2) subjective complaints participants made (e.g., the presence/absence of gastrointestinal discomfort), and (3) overall adherence determined by the self-reported number of drinks consumed (total of 42), as well as through serum analysis for BCAA concentration.

#### Sleep

Outcomes related to sleep quality included self-report and wrist based actigraphy in conjunction with daily sleep diary entries. Self-reported outcomes specific to sleep consisted of the Insomnia Severity Index (ISI) and the Sleep Hygiene Index (SHI). Together these assessments probe general difficulty in initiating and maintaining sleep, as well as common behavioral habits that contribute to sleep disruption. The ISI is a 7-item measure, each item being a 5-point Likert scale producing a total score ranging from 0 to 28 (Morin, 1993; Bastien et al., 2001). The SHI is a 13-item measure, each item being a 5-point Likert scale, producing a total score ranging from 0 to 52 (Mastin et al., 2006).

Wrist actigraphy was collected in 2-min epochs continuously for the full 28-day testing duration (7 days of baseline and 21 days of dietary supplementation). Participants wore their actiwatch on their non-dominant wrist and were instructed and reminded throughout the study to be cognizant of not allowing clothing to cover the photosensor. Actigraphy data were analyzed using the Actiware version 6.0.9 proprietary algorithm (Philips Respironics, Bend, OR, United States) with the activity threshold set to “medium.” Each study day, from 12:00 p.m. to 11:58 a.m. was analyzed individually for bedtime, sleep onset, wake time, mid-sleep time, total sleep time (TST), time in bed (TIB), sleep onset latency (SOL), sleep efficiency (SE), wake after sleep onset (WASO), total activity, average activity/epoch, and number of nocturnal awakenings. Actigraphy metrics came from the final 3–5 days of the baseline period and the final 3–5 days of the BCAA supplementation period. Participants were also instructed to keep daily sleep diaries to record times in/out of bed. To minimize heterogeneity within subjects, sleep diaries were examined for days where subjects reported not working, working aberrant schedules, or when ill or traveling; these periods were excluded from analyses. On average, this sleep diary assessment resulted in excluding 1–2 days over the 28-day study period per subject.

### Exploratory Outcomes

#### Cognition

Participants engaged in a series of standard neuropsychological tests to assess various cognitive domains, including attention/executive function, language, short/long term memory, and visuospatial function. The following tests were administered by trained study personnel: Controlled Oral Word Association Test (COWAT), Hopkins Verbal Learning Test—Revised (HVLT-R), the Wechsler Adult Intelligence Scale-IV (WAIS-IV; digit span, symbol search, and letter-number sequencing), and Delis-Kaplin Executive Function System (D-KEFS; color-word interference).

Additionally, self-reported cognition was assessed *via* the NIH PROMIS Cognitive Function short form is 4-items, each 5-point Likert scales, that broadly assess self-reported metrics of executive function, attention, and short-term memory.

#### Pain

Current pain was assessed *via* self-report and semi-objectively through pressure algometry. Self-reported current pain over the past 7–14 days was assessed *via* a standard 11-point Likert scale as well as the NIH PROMIS Pain Interference and Pain Intensity short-form scales (Cella et al., 2007; Hays et al., 2009).

Somatic pressure pain threshold and tolerance testing using hand-held pressure algometry (AlgoMed; Medoc, Ramat Yishai, Israel) was measured over the lunula of the thumb on participant’s dominant side. Algometry was performed by exerting a constantly increasing rate of pressure (kPa), perpendicular to the participant, with the aid of computer interface plotting real-time pressure. Participants used a handheld button that produces a tone when pressed to signal the pressure they perceive that corresponded to their pressure pain threshold (i.e., perception of initial pain/discomfort) and tolerance (i.e., perception of pain/discomfort resulting in a desire to withdraw their hand). Both threshold and tolerance were conducted across three trials, averaging the second and third trial, separated by 2 min. The algometer was calibrated before each participant, room temperature was thermoneutral at 22 ± 2°C, and the ambient light intensity was ∼100 lux (i.e., dim light conditions). The same investigator conducted all pressure pain testing (Chesterton et al., 2007).

#### Blood-Based Inflammatory Biomarkers

Whole blood was collected pre- and post-intervention (i.e., generally day 1 and 28 of the protocol, corresponding to the beginning of baseline and end of intervention) *via* an antecubital vein, immediately inverted 10x and stored at 4°C until processing for plasma (lavender top ethylenediaminetetraacetic vacutainer) and serum (red/gray “tiger” top vacutainer). The average delay from collection to processing was ∼1–3 h. Aliquots were stored at −80°C until enough were obtained for batch assays. Serum samples were assayed for BCAA content by the OHSU Oregon Clinical and Translational Research Institute (OCTRI) Core Lab using a standard assay.

Plasma samples were sent to the NIH NINR Biomarker Laboratory (PI: JMG) for analysis (Mondello et al., 2014; Korley et al., 2019; Edwards et al., 2020; Guedes et al., 2020). The Meso Scale Discovery (MSD) V-Plex Proinflammatory Panel 1 Human Kit (Rockville, Maryland, United States) was used to measure plasma interleukin (IL)-6, IL-8, IL-10, and tumor necrosis factor alpha (TNF-α) concentrations in single samples, according to the manufacturer’s instructions. The lower limit of quantification and detection for each kit were determined from the respective standard curves for each cytokine. The reported coefficient of variation (intraplate and interplate) for MSD values were no higher than 20% for all analytes.

#### Questionnaires

Additional exploratory self-reported outcomes included assessments of quality of life, neurotrauma-related symptom severity, and mood/anxiety. Quality of life metrics were obtained through the World Health Organization Disability Rating Scale (WHO-DAS 2.0) and the Neuro QoL v1.0 Participation and Satisfaction short form measures. Neurotrauma-relevant measures consisted of the PTSD checklist for DSM-5 (PCL-5), enabling a provisional PTSD diagnosis (Blevins et al., 2015; Balba et al., 2018; Elliott et al., 2018b,^2020^) and the Neurobehavioral Symptom Inventory (NSI) for post-concussive sequela (Belanger et al., 2009; Bulson et al., 2012; Vanderploeg et al., 2014). Mood/depression and anxiety were assessed through the Patient Health Questionnaire (PHQ-9) and the NIH PROMIS Emotional Distress-Anxiety (EDA) scale.

### Statistical Analysis

Analyses were performed using GraphPad Prism (v.9). An alpha value of 0.05 (defined *a priori*) was used for all tests, unless otherwise noted. Mean differences between pre- and post-intervention outcomes within BCAA, rice protein, and microcrystalline cellulose groups were assessed *via* paired two-tailed students *t*-test or chi-square test, where appropriate.

## Results

### Demographic, Medical, and Military Service History

Demographic data for participants are shown in Table 1. There were no significant differences between groups regarding sex, age, BMI, race, years of education, average duration of weekly exercise, or in metrics related to TBI history. All reported TBIs were classified as mild injuries. Medical history was obtained *via* self-report, with no significant differences in participants medical history, including cancer, cardiovascular health, autonomic management, pain syndromes, neurotrauma history, cognitive complaints, and current medication usage for sleep, pain, or depression. Military service history was also collected *via* self-report, with no significant differences in service history, including duration of service, combat exposure, percent service connection, branch of service, and total number of deployments.

**TABLE 1:** Demographic information across all participants.

	BCAA (*n* = 9)	Rice (*n* = 8)	Cellulose (*n* = 9)
Sex, female	2 (22.2%)	4 (50.0%)	2 (22.2%)
Age, years	47.7 ± 11.4	49.3 ± 10.6	50.7 ± 6.6
BMI, kg/m^2^	32.1 ± 3.1	34.8 ± 5.4	37.3 ± 3.9
Race, white	9 (100%)	7 (87.5%)	5 (55.6%)
Education, ≥some college	4 (44.4%)	2 (25.0%)	4 (44.4%)
Exercise, ≥90 min/week	5 (55.6%)	3 (37.5%)	5 (55.6%)
TBI characteristics			
Years post-injury	17.0 ± 8.3	22.6 ± 9.4	34.0 ± 6.7
Age of injury, years	32.1 ± 15.1	17.38 ± 9.6	23.6 ± 7.4
Number of injuries	2.8 ± 2.4	6.1 ± 7.1	3.6 ± 3.78

*Data are presented as n (% total) or mean ± standard deviation. The rice protein and cellulose groups both included n = 1 transgender woman. BCAA, branch chain amino acid; BMI, body mass index. Other Race, Rice: n = 1 Black or African American; Other Race, Cellulose: n = 1 American Indian/Alaska Native and n = 3 not reported. Continuous variables were analyzed via one-way ANOVA with Tukey post-hoc comparison, and categorical variables via Chi-square with Bonferroni post-hoc comparison.*

### Primary Outcomes

#### Acceptability, Feasibility, and Adherence

Of the 32 participants consented, each participant underwent block randomization, stratified by age (Cerra et al., 1983; Horst et al., 1984; Smith et al., 1987; Robertson et al., 1988; Skeie et al., 1990; Blomstrand et al., 1991; Wagenmakers, 1992; Plauth et al., 1993; Hasseman et al., 1994; Cangiano et al., 1996; Manner et al., 1996; Tandan et al., 1996; Yudkoff, 1997; Richardson et al., 1999; Blomstrand, 2001; James, 2002; Mori et al., 2002; Marchesini et al., 2003; Scarna et al., 2003; Dijkers, 2004; Mahmood et al., 2004; Moldover et al., 2004; Aquilani et al., 2005; Fernstrom, 2005; Matthews, 2005; Hawkins et al., 2006; Vasterling et al., 2006; Worthington and Melia, 2006; Ozgultekin et al., 2008; Cifu et al., 2009; Lew et al., 2009; Bombardier et al., 2010; Cole et al., 2010; Scholten et al., 2012; Lim et al., 2013; Wiseman-Hakes et al., 2013; Corrigan et al., 2014; Rao et al., 2014; Elkind et al., 2015; Boakye et al., 2016; Knapik et al., 2016; Nguyen et al., 2017; Elliott et al., 2018a,^2021^; Edwards et al., 2020; Pattinson et al., 2020; Peltz et al., 2020) and sex, to one of three experimental arms (Figure 1). Two participants from each of the three experimental arms withdrew after randomization. The two participants in the rice protein group withdrew due to discomfort from wearing the actiwatch, with no complaints about the supplementation itself. One of BCAA group participants withdrew due to disliking the actiwatch, while the other withdrew due to poor taste from the supplement (taste alone; no gastrointestinal discomfort). The two participants in the microcrystalline cellulose group withdrew based on study burden/time constraints.

Post-treatment surveys given to each group demonstrated adequate acceptability of the supplement and actiwatches. Of the 26 participants who completed the study, 8 (31%) reported they would continue taking the supplement if given the opportunity, 5 (19%) reported not liking the supplement, and 13 (50%) neither liked nor disliked the supplement. The primary complaint for those who did not like the supplement was that they experienced upset generalized gastrointestinal discomfort (e.g., mild stomachache and nausea) or disliked the taste/texture. The BCAA group had more complaints of generalized gastrointestinal discomfort (*n* = 4) but also reported better sleep outcomes, specifically relating to less sleep disturbances (*n* = 3) and said they would continue if given the opportunity. The microcrystalline cellulose group mainly reported not noticing a difference (*n* = 5), with no positive sleep outcomes reported. Finally, participants in the rice group were mixed, some saying they felt better overall and would continue given an opportunity (*n* = 3), while others noticed no difference (*n* = 3). The actiwatch was well-accepted with 16 (62%) of the participants experienced no issues in wearing the actiwatch, and although 9 (35%) found it uncomfortable, adherence was complete across all subjects.

**FIGURE 2:**
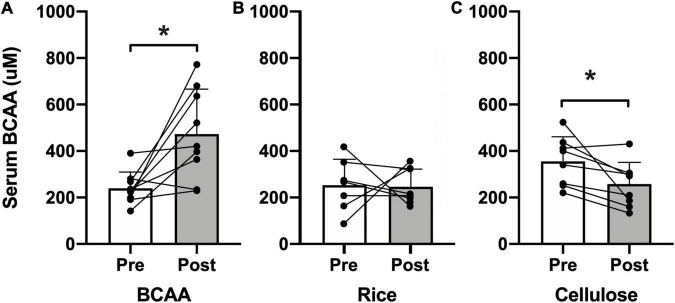
Serum BCAA concentration. Pre- (open bars) and post-intervention (shaded bars) serum BCAA concentration (μM) for the **(A)** BCAA, **(B)** rice protein, and **(C)** microcrystalline cellulose groups (mean ± standard deviation). Individual data points with connecting lines overlaid. *p < 0.05, paired two-tail *t*-test.

Treatment fidelity and adherence were objectively assessed by examining serum BCAA concentration pre- and post-intervention (Figure 2). All three groups reported normal levels of serum BCAA concentrations at baseline (BCAA, 239.6 μmol/L; rice protein, 252.8 μmol/L; microcrystalline cellulose, 355.6 μmol/L). Only participants in the BCAA group demonstrated a significant rise in serum BCAA concentration post-intervention (472.7 μmol/L; *p* = 0.010), compared to the rice protein (246.0 μmol/L, *p* = 0.968) and microcrystalline cellulose groups (258.0 μmol/L, *p* = 0.028).

#### Sleep

**FIGURE 3:**
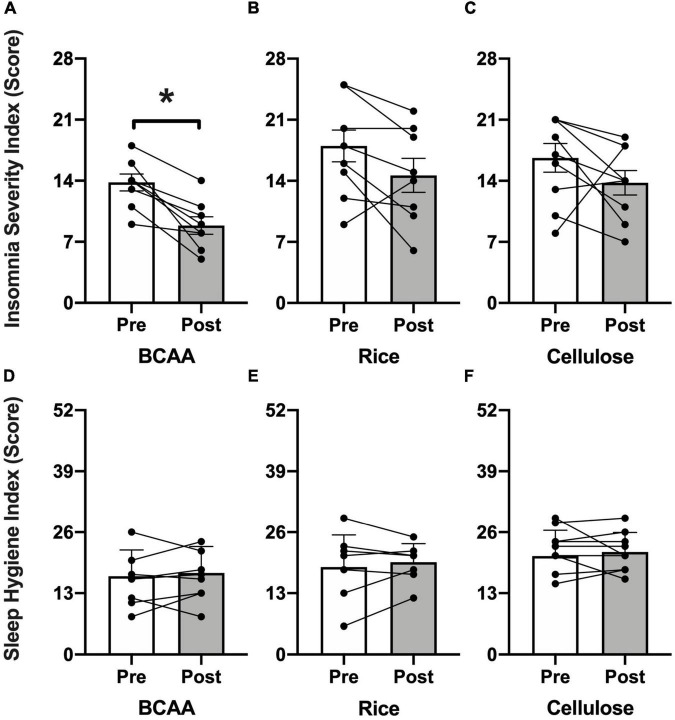
Insomnia Severity Index (ISI) and Sleep Hygiene Index (SHI). Pre- (open bars) and post-intervention (shaded bars) ISI scores (range = 0–28; higher = increased impairment) for the **(A)** BCAA, **(B)** rice protein, and **(C)** microcrystalline cellulose groups with SHI scores (range = 0–52; higher = worse sleep hygiene) for the **(D)** BCAA, **(E)** rice protein, and **(F)** microcrystalline cellulose groups (mean ± standard deviation). Individual data points with connecting lines overlaid. *p < 0.05, paired two-tail *t*-test.

Self-reported sleep (Figure 3) as assessed by the ISI improved in the BCAA group (13.4 ± 2.7 to 11.9 ± 5.5; *p* < 0.002) and rice protein group (17.5 ± 5.7 to 14.8 ± 4.1; *p* < 0.005), but no change in the microcrystalline cellulose group (17.3 ± 3.8 to 12.7 ± 4.2; *p* = 0.10). There was no change in sleep hygiene as assessed by the SHI in any of the 3 groups (BCAA: 15.8 ± 5.6 to 17.3 ± 5.6; *p* = 0.62; rice: 18.9 ± 7.5 to 19.6 ± 3.9; *p* = 0.69; microcrystalline cellulose: 22.4 ± 4.6 to 21.8 ± 4.2; *p* = 0.59). This lack of change in sleep hygiene is supportive of the premise that observed sleep effects are not explained simply by changes in behavior or routine.

**FIGURE 4:**
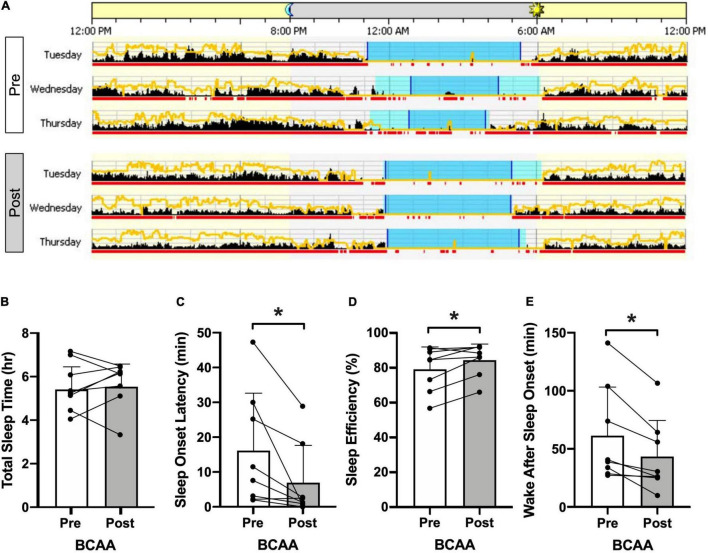
Actigraphy. **(A)** Representative pre- and post-intervention 3 consecutive mid-week 24-h periods for the BCAA group produced using Philips Actiware. The yellow line represents ambient light exposure and black columns reflect activity counts in 2-min bins. Sleep and wake is differentiated by the red underline (wake) and the blue shaded period indicates the entire “rest interval” with time in bed resting but not asleep) being the light blue intervals and time in bed asleep being the dark blue intervals. Specific actigraphy metrics are plotted in panels **(B)** total sleep time, **(C)** sleep onset latency, **(D)** sleep efficiency, and **(E)** wake after sleep onset, in the BCAA group with pre- and post-intervention being open and shaded bars (individual data overlaid). *p < 0.05, paired two-tail *t*-test.

Actigraphy outcomes (Figures 4A,B and Table 2) were consistent with the self-reported improvement in insomnia. Representative actigrams are shown for three consecutive days pre- and post-intervention in the BCAA group (Figure 4A). The BCAA group showed an average decrease in sleep onset latency by ∼9-min (16.1 ± 16.5 to 6.9 ± 10.7; *p* < 0.03) (Figure 4C), which corresponded with an improvement in sleep efficiency by ∼5% (79.4 ± 12.8 to 84.6 ± 9.2; *p* < 0.03) (Figure 4D). This also led to an improvement in wake after sleep onset by a decrease of ∼18-min (61.2 ± 41.8 to 43.1 ± 31.2; *p* < 0.008) (Figure 4E). Of note, total sleep time remained unchanged, which is consistent with participant’s time in bed, bedtime, wake time, and mid-sleep time remaining consistent throughout the study.

**TABLE 2:** Actigraphy.

	BCAA		Rice		Cellulose	
	Pre	Post	Pre	Post	Pre	Post
Bedtime, hh:mm	23:35 ± 1:21	23:39 ± 1:32	23:52 ± 2:05	23:43 ± 2:20	23:11 ± 1:14	22:44 ± 0:48
Waketime, hh:mm	6:31 ± 2:05	6:47 ± 2:21	7:57 ± 2:33	7:28 ± 2:33	6:46 ± 1:43	6:33 ± 2:31
Mid-sleep time, hh:mm	3:03 ± 1:36	3:14 ± 1:55	3:55 ± 2:15	3:14 ± 2:26	2:59 ± 1:26	2:39 ± 1:23
Time in bed, hh:mm	7:03 ± 1:24	7:17 ± 1:03	8:07 ± 0:43	8:06 ± 0:45	7:35 ± 1:13	7:48 ± 1:09
TST, hh:mm	5:41 ± 1:05	5:53 ± 1:04	6:07 ± 1:42	6:06 ± 1:24	6:37 ± 1:09	6:39 ± 1:13
SOL, min	16.1 ± 16.5	6.9 ± 10.7*	11.4 ± 10.4	14.7 ± 15.9	6.9 ± 8.4	5.4 ± 6.1
SE, min	**79.4 ± 12.8**	84.6 ± 9.2*	75.1 ± 17.4	76.0 ± 18.0	87.2 ± 4.6	86.1 ± 6.4
WASO, min	**61.2 ± 41.8**	43.1 ± 31.2*	84.7 ± 50.0	75.0 ± 40.3	**40.6 ± 14.7**	50.0 ± 15.8*
# Awakenings	14.7 ± 5.6	12.0 ± 6.4	15.8 ± 4.8	15.0 ± 3.2	13.4 ± 4.1	13.0 ± 4.3

Data are mean ± SD. TST, total sleep time; SOL, sleep onset latency; SE, sleep efficiency; WASO, wake after sleep onset. Data were analyzed via paired students t-test within each group. *p < 0.05 vs. Pre.

### Exploratory Outcomes

#### Cognition

Overall, no significant effects were observed pre- vs. post-intervention within this battery of cognitive function assessments. However, for the purposes of completeness, all neuropsychological testing results are described below. The COWAT-FAS and categorical fluency (animals), measures of language and executive function, pre-intervention raw scores averaged 35 ± 10 (scaled scores = 10 ± 3) and 22 ± 5 (scaled scores = 11 ± 3), respectively. Post-intervention, both COWAT-FAS and categorical fluency measures increased (non-significantly) by ∼10%. Pre- and post-intervention the Hopkins Verbal Learning Test—Revised (HVLT-R; measure of short-term memory) showed raw total recall scores (sum of three trials) averaging 23 ± 4, retention rates of 85–90 ± 10%, and raw retention discrimination indexes of 10 ± 2. The Wechsler Adult Intelligence Scale-IV (WAIS-IV; measure of attention/executive function and visuospatial function) demonstrated consistent reliability across participants with no evidence for lack of effort, intention or otherwise (the digit span, symbol search, and letter-number sequencing subtests were used). Total digit span scores (forward + backward + sequencing) were 27 ± 5 pre- to post-intervention across groups. Symbol search raw scores pre- to post-intervention in the BCAA group increased (non-significantly) from 32 ± 10 to 38 ± 8 (*p* = 0.07), with similar pre-intervention scores of 33 ± 8 in the rice protein and cellulose groups. Letter-number sequencing raw scores were 18 ± 3 pre- to post-intervention across groups. Lastly the Delis-Kaplin Executive Function System (D-KEFS; measure of attention/executive function and visuospatial function) also showed non-significant changes across groups. For example, the color-word interference subtest inhibition raw and inhibition-switching trials pre- to post-intervention in the BCAA group changed from 55 ± 9 to 67 ± 24 (*p* = 0.08), and 66 ± 16 to 72 ± 20 (*p* = 0.07), respectively.

Additionally, self-reported cognition was assessed *via* the NIH PROMIS Cognitive Function short form is 4-items, each 5-point Likert scales, that broadly assess self-reported metrics of executive function, attention and short-term memory. Again, no significant changes in self-reported cognitive function were observed. Pre- to post-intervention scores averaged 12–15 ± 5 across groups.

#### Pain

Self-reported current pain intensity, derived from a standard 10-point Likert scale assessing average pain over the past week, decreased (non-significantly) in the BCAA group from 5.1 ± 1.8 to 4.0 ± 1.7 (*p* = 0.07) post-intervention, while the rice protein (5.1 ± 2.8 to 4.9 ± 3.3; *p* = 1.0) and microcrystalline cellulose (4.9 ± 2.5 to 4.3 ± 2.8; *p* = 0.351) groups showed smaller absolute changes. Quantitative pain assessments, done *via* pressure algometry, showed an improvement in pressure-pain tolerance in the BCAA group (550.1 ± 223.0 to 642.7 ± 263.9 kPa; *p* = 0.049) with no change in pressure-pain threshold (329.9 ± 201.6 to 453.4 ± 230.7 kPa; *p* = 0.07). No changes in quantitative pressure-pain assessments were found in the rice protein and microcrystalline cellulose groups.

#### Blood-Based Inflammatory Biomarkers

Changes in plasma biomarkers related to peripheral markers of inflammation were assessed *via* MSD based ultrasensitive ELISA. The BCAA group demonstrated a significant reduction in the pro-inflammatory cytokine TNF-α (0.89 ± 0.30 to 0.73 ± 0.29; *p* = 0.03) and a significant increase in the anti-inflammatory cytokine IL-10 (0.27 ± 0.06 to 0.38 ± 0.11; *p* = 0.04), without changes in IL-6 (*p* = 0.48) or IL-8 (*p* = 0.26). The rice protein and cellulose groups both showed a significant reduction in IL-10 (rice: 0.34 ± 0.16 to 0.25 ± 0.18, *p* = 0.006; cellulose: 0.19 ± 0.07 to 0.15 ± 0.08, *p* = 0.026), with no changes in IL-6, IL-8 or TNF-α.

#### Additional Self-Reported Measures

Participants also completed a battery of self-report measures, assessing neurotrauma symptom severity (*via* the Neurobehavioral Symptom Inventory, or NSI, for TBI and the Posttraumatic stress disorder Checklist 5, or PCL-5, for PTSD), anxiety (*via* the NIH PROMIS Emotional Distress and Anxiety short-form 3a), depression (*via* the Patient Health Questionnaire-9), quality of life (*via* the World Health Organization Disability Assessment Scale 2.0 and the NeuroQoL participation and satisfaction measures). No statistically significant group differences were identified in any of these measures.

## Discussion

The present study is a randomized double-blind placebo-controlled pilot and feasibility trial that demonstrates dietary supplementation with BCAAs is feasible, acceptable, and improves both subjective and objective sleep outcomes in Veterans with TBI. Furthermore, exploratory outcomes of interest showing potential for improvement includes blood-based markers of systemic inflammation, subjective pain and semi-quantitative nociception, and neuropsychological testing of cognition. This is the first study to translate the known effect of BCAA supplementation to improve sleep in rodent models of TBI to humans with TBI, and supports rationale for a full-scale efficacy trial.

The BCAA intervention showed high feasibility and high acceptability, with <20% dropout spread evenly across treatment and placebo groups. Over 95% of participants were fully adherent across 21-days of dietary BCAA supplementation (30 g, b.i.d.) and reported minimal subjective complaints with no adverse events. High treatment fidelity and adherence, as assessed by elevated serum BCAA concentrations, showed objective evidence of protocol adherence.

The BCAA intervention showed preliminary efficacy for BCAA supplementation to improve both subjective and objective sleep as assessed by self-report and wrist actigraphy outcome measures. BCAA supplementation also showed significant improvements in exploratory outcome measures, including blood-based inflammatory markers, self-reported pain and semi-quantitative pressure algometry, and potential improvements in certain neuropsychological parameters (e.g., attention/executive function and visuospatial domains). Taken together, these data support the rationale and inform the design of a large scale randomized clinical trial to definitively assess the efficacy of BCAAs to improve sleep and related secondary outcome measures.

### Human Safety Studies

Previous work in humans has explored the therapeutic use of BCAAs in improving health and minimizing disease. BCAAs have been used in athletes to improve physical performance and mental concentration/focus during training and competition (Hasseman et al., 1994; Blomstrand, 2001). The rationale for this use is that prolonged or intense exercise causes depletion of BCAAs within (a) the muscle, negatively impacting muscle bioenergetics and accelerating peripheral fatigue and (b) the plasma, result in an increase in CNS uptake of tryptophan, synthesis of serotonin, and promotion of central fatigue. Dietary BCAA supplementation has also been used in patients with hepatic cirrhosis, demonstrating its safety in the presence of liver dysfunction (Horst et al., 1984; Plauth et al., 1993; James, 2002). Because hepatic cirrhosis causes elevated levels of similar competing amino acids (e.g., tryptophan, tyrosine, and phenylalanine) due to a reduced ability to metabolize these amino acids, raising plasma BCAA concentrations has been shown to help normalize CNS neurotransmitter composition (Plauth et al., 1993; James, 2002). Finally, BCAAs have been studied in a variety of psychiatric and neurological disorders including as a treatment for patients with bipolar disorder (by reducing tyrosine uptake and preventing manic episodes) (Scarna et al., 2003), schizophrenia with impaired motor control (by reducing the relative concentration of CNS phenylalanine concentrations) (Richardson et al., 1999), amyotrophic lateral sclerosis (by restoring cortical E:I balance) (Tandan et al., 1996), spinocerebellar degeneration (Mori et al., 2002), and cancer (to stimulate anabolism and prevent muscle wasting) (Cerra et al., 1983; Cangiano et al., 1996).

There has been limited work in treating humans with TBI with BCAA supplementation. These studies have predominantly been in the context of severe TBI (i.e., TBI requiring hospitalization) but nevertheless have reported that BCAA supplementation reduced plasma BCAA levels in the acute post-injury period (Aquilani et al., 2003; Vuille-Dit-Bille et al., 2012) and up to 17 months post-injury (Borsheim et al., 2007), improved cognition post-injury (Aquilani et al., 2005, 2008), and improved cerebral oxygen consumption (Vuille-Dit-Bille et al., 2012). In addition to this published work, there is one ongoing clinical trial (HITHEADS; NCT01860404) exploring the potential for dietary BCAA supplementation to improve cognition after mild, sports-related TBI in young adults (ages 11–34) in the acute phase after TBI (<72 h post-TBI). Our current population, e.g., Veterans who are in the chronic phase of recovery from TBI, fills a critical knowledge gap in the existing literature of BCAA supplementation for TBI-related outcomes.

### Proposed Mechanism of Action of Branched Chain Amino Acid

Over the past decade, our group has conducted extensive preclinical studies of dietary BCAA supplementation on both sleep and cognition using a well-characterized mouse model of mTBI. With regard to sleep, initial results in mice with TBI showed that persistent sleep-wake disturbances were ameliorated by BCAA therapy (Lim et al., 2013); a finding supported by the present study in humans. Later it was demonstrated that BCAA’s mechanism of action was to restore decreased glutamate levels in presynaptic terminals contacting orexin/hypocretin neurons in the hypothalamus (Elliott et al., 2018a). Orexin/hypocretin is a neuropeptide that controls the stability of sleep and wake states; this molecule is deficient in human narcolepsy and also decreased in human cerebral spinal fluid after TBI (Mahoney et al., 2019). Previous work has shown that sleep-wake regulation is significantly impaired in two different preclinical models of TBI (i.e., controlled cortical impact and fluid percussion injury in mice) (Lim et al., 2013; Elliott et al., 2018a). Concurrent with sleep impairments, we have shown a reduction in orexin/hypocretin neuronal activation (Mori et al., 2002), a finding that is consistent with other work examining orexin/hypocretin cell counts in mice (Willie et al., 2012) and rats (Skopin et al., 2015). Importantly, dietary BCAA supplementation restored normal sleep-wake regulation and orexin/hypocretin neuronal activation (Lim et al., 2013). Furthermore, recent work employing a novel immuno-gold double labeling approach to visualize glutamate levels within presynaptic nerve terminals with electron microscopy in a mouse model of TBI, has shown dietary BCAA supplementation restored glutamate levels within presynaptic nerve terminals contacting orexin/hypocretin neurons (Elliott et al., 2018a). These data support previous work connecting dietary BCAA supplementation driving improved outcomes with increased synaptic efficacy (Cole et al., 2010) and neuronal activity (Lim et al., 2013) by way of increased glutamate within the pre-synaptic nerve terminals (Elliott et al., 2018a).

With regard to cognition, dietary BCAA supplementation reduced mTBI-induced cognitive impairment, restored normal plasma and hippocampal BCAA levels, and improved net synaptic efficiency within CA1 of the hippocampus (Cole et al., 2010). Our subsequent work extended these findings by confirming mTBI-induced behavioral impairments and cognitive deficits stemmed from a disrupted balance between excitatory and inhibitory cortical synaptic transmission within prefrontal cortex (Smith et al., 2015). In summary, our preclinical studies elucidate the neural mechanism by which BCAAs restore network excitability and inputs to sleep and cognitive areas of the brain.

Previous work by our group in mice with TBI has demonstrated that TBI causes reduced BCAA levels within the hippocampus, a brain structure involved in higher-order cognitive function (Cole et al., 2010). In these mice, BCAA supplementation returned hippocampal BCAA to pre-TBI levels (Cole et al., 2010). Furthermore, deficits in hippocampal CA1 net synaptic efficacy, which correlates with deficits in hippocampal-dependent contextual fear conditioning, were restored after BCAA supplementation (Cole et al., 2010). Later work extended these findings by examining the excitatory and inhibitory balance between cortical synaptic transmission within prefrontal cortex after TBI (Smith et al., 2015). Finally, our more recent work has shown that mice with TBI show specific deficits in spatial episodic memory which are improved by BCAA therapy (Paterno et al., 2018).

### Branched Chain Amino Acid Dosing, Duration, and Administration

Several rodent and human studies have shown that TBI is associated with reduction in plasma BCAA levels (post-injury range: acute to 17 months) (Aquilani et al., 2003, 2005; Borsheim et al., 2007; Vuille-Dit-Bille et al., 2012; Jeter et al., 2013; Sharma et al., 2018) resulting from significant BCAA efflux from a hypermetabolic and hypercatabolic phenotype (comparable to severe burn patients) (Ott et al., 1994). Acute (Borsheim et al., 2007; Vuille-Dit-Bille et al., 2012) and sustained BCAA administration (Aquilani et al., 2005, 2008; Ozgultekin et al., 2008) restored plasma BCAA to pre-injury levels. Additionally, previous work has shown sustained BCAA administration (range: intravenous 19.6 g/day for 15 days or oral 30 g/day for 10 days) improves cognitive outcomes (Aquilani et al., 2005, 2008; Ozgultekin et al., 2008). In 8 previous studies utilizing acute doses of oral BCAAs ranging from 5 to 90 g, no adverse events were reported, and it was universally noted that the drinks containing BCAAs were well-tolerated (Blomstrand et al., 1991; Hasseman et al., 1994; van Hall et al., 1995; Kirvela et al., 1998; Struder et al., 1998; De Palo et al., 2001; Gijsman et al., 2002). Extensive work has also been done employing chronic BCAA administration. These studies had doses ranging from 15 to 60 g/day and durations between 7 days and 12 months (c.f. Fernstrom, 2005, 2013). Only one study reported mild gastrointestinal discomfort (Marchesini et al., 2003); all others indicated that the BCAA interventions were well-tolerated.

In our preclinical mouse studies, we determined the dose and duration of dietary BCAA supplementation needed to restore cognitive impairment (Elkind et al., 2015). Mice were given *ad libitum* access to BCAA supplemented drinking water (100 or 50 mM; average consumption 3–5 ml/day), or BCAA administration *via* oral gavage (0.26 g/kg). Freezing behavior after contextual fear conditioning (a measure of memory formation) was assessed over 10 days (Elkind et al., 2015). We showed that BCAA administration was required for at least 5 days and at 100 mM to restore contextual fear conditioning behavior (BCAA 100 mM dosing in mice is equal to 0.26 g/kg, which is equivalent to ∼20–30 g/day in a 70–90 kg human).

With regard to the doses chosen for this study (60 g/day, divided into b.i.d. dosing), previous work in rats has determined the safe upper limit of leucine consumption is 8.9 g/kg bodyweight (equivalent to ∼600 g leucine/day in a 70 kg human), an approximately 10-fold higher dose that our minimum effective dose in mice (Sakai et al., 2004; Pencharz et al., 2008). Along the same lines, HITHEADS is administering doses between 15 and 60 g/day of BCAA supplementation to acutely concussed adolescents. For these reasons, we chose to use 60 g/day of BCAA for 21 days (NCT03990909). Further work is needed to determine efficacy of lower doses and longer duration of BCAA supplementation in individuals in the chronic, post-concussive phase of recovery from TBI.

### Limitations

Although this study has strengths in that it was randomized, double-blind, and placebo-controlled, it is still a pilot and feasibility trial with respect to statistical power. Sample sizes were not powered for efficacy, and thus despite comparisons reaching statistical significance in key outcome variables, interpretations should still be cautious. For example, while TBI variables such as number of prior injuries did not significantly differ across groups, their mean values still may be clinically relevant: The Rice protein group reported an average of 6.1 TBIs, whereas the BCAA group reported an average of 2.8 TBIs (with the increased number of TBIs in the Rice group driven primarily by a single participant who reported 22 TBIs). As there is known clinical and biological relevance in the number of prior TBIs, this variance between groups may represent a potential confound in interpretation of our results.

A second limitation is that the dropout rate was 18%, which will be used to inform future clinical trial design by increasing sample sizes accordingly and/or addressed with increased coordinator engagement, subject incentives, etc. Similarly, the timeline for this intervention was 3 weeks in duration, consistent with the short duration to effect seen in preclinical studies—however, this relatively short timeframe may not be sufficient to show robust cognitive effects in humans. Future clinical trial design will consider a longer intervention duration to improve the sensitivity for detecting a change in secondary outcomes. Specific to the BCAA group, palatability was a commonly heard issue, and will need to be addressed by collaboration with food and flavor chemists. The current study used block randomization to balance groups for age and sex, but the future full-scale trial will consider other factors besides age/sex that could inadvertently drive group differences, including TBI recency, pain level, depression/PTSD status. These other factors will be incorporated into the larger trial using a covariate adaptive randomization scheme. Finally, specific to sleep outcomes, the future full-scale trial will incorporate pre- and post-intervention overnight polysomnography in order to assess gold standard sleep staging and other electroencephalography-based metrics relevant to TBI (Modarres et al., 2017, 2019).

## Conclusion

Dietary supplementation with BCAA is a mechanism-based, promising intervention that shows feasibility, acceptability, and preliminary efficacy to treat insomnia and objective sleep disruption in Veterans with TBI. A larger scale randomized clinical trial is warranted to further evaluate the efficacy, dosing, and duration of BCAA effects on sleep and other related outcome measures in individuals with TBI.

## Data Availability Statement

The datasets presented in this study can be found in online repositories. The names of the repository/repositories and accession number(s) can be found in the article/supplementary material.

## Ethics Statement

The studies involving human participants were reviewed and approved by VA Portland Health Care System. The patients/participants provided their written informed consent to participate in this study.

## Author Contributions

JE, AC, and ML: conception and design of the study. JE, AK, MO’N, SM, JG, and ML: acquisition and analysis of data. JE and ML: drafting a significant portion of the manuscript or figures (i.e., a substantial contribution beyond copy editing and approval of the final draft, which was expected of all authors).

## Author Disclaimer

The views expressed in this article are those of the authors and not necessarily those of the NHS, the NIHR, or the Department of Health and Social Care.

## Conflict of Interest

The authors declare that the research was conducted in the absence of any commercial or financial relationships that could be construed as a potential conflict of interest.

## Publisher’s Note

All claims expressed in this article are solely those of the authors and do not necessarily represent those of their affiliated organizations, or those of the publisher, the editors and the reviewers. Any product that may be evaluated in this article, or claim that may be made by its manufacturer, is not guaranteed or endorsed by the publisher.

## Abbreviations

BCAA: branched chain amino acid
b.i.d.: twice daily
BMI: body mass index
COWAT: Controlled Oral Word Association Test
CNS: central nervous system
D-KEFS: Delis-Kaplin executive function system
ELISA: enzyme linked immunosorbent assay
FOSQ-10: Functional Outcomes of Sleep Questionnaire-10
GABA: gamma-aminobutyric acid
HTEC: head trauma events characteristics
HVLT-R: Hopkins verbal learning test—revised
ISI: insomnia severity index
IL: interleukin
LOC: loss of conscious
NIH PROMIS: National Institute of Health Patient Reported Outcome Measurement Information System
NSI: neurobehavioral symptom inventory
PCL-5: post-traumatic stress disorder checklist for DSM-5
PHQ-9: Patient Health Questionnaire-9
PTSD: post-traumatic stress disorder
SE: sleep efficiency
SHI: sleep hygiene index
SOL: sleep onset latency
TBI: traumatic brain injury
TIB: time in bed
TST: total sleep time
TNF-α: tumor necrosis factor alpha
WAIS-IV: Wechsler Adult Intelligence Scale-IV
WASO: wake after sleep onset
WHO-DAS 2.0: World Health Organization Disability Rating Scale 2.0.
